# How Have Intravitreal Anti-VEGF and Dexamethasone Implant Been Used in Italy? A Multiregional, Population-Based Study in the Years 2010–2016

**DOI:** 10.1155/2020/7582763

**Published:** 2020-01-09

**Authors:** Giulia Scondotto, Janet Sultana, Valentina Ientile, Ylenia Ingrasciotta, Andrea Fontana, Massimiliano Copetti, Eliana Mirabelli, Costantino J. Trombetta, Carlo Rapisarda, Michele Reibaldi, Teresio Avitabile, Antonio Longo, Patricia Ibanez Toro, Maria Vadalà, Salvatore Cillino, Gianni Virgili, Rosa Gini, Olivia Leoni, Sebastiano Walter Pollina Addario, Pasquale Cananzi, Claudia La Cavera, Maria Rosalia Puzo, Giovambattista De Sarro, Adele De Francesco, Gianluca Trifirò

**Affiliations:** ^1^Unit of Clinical Pharmacology, A.O.U. “G. Martino”, Messina, Italy; ^2^Department of Biomedical and Dental Sciences and Morpho-functional Imaging, University of Messina, Messina, Italy; ^3^Unit of Biostatistics, Fondazione IRCCS “Casa Sollievo Della Sofferenza”, San Giovanni Rotondo FG, Italy; ^4^Department of Ophthalmology, A. O. U. Policlinic-Vittorio Emanuele, Catania, Italy; ^5^Assistance and Pharmaceutical Services Office, Personal Policies Department, Basilicata Region, Potenza, Italy; ^6^Institute of European and Mediterranean Science and Technology (IEMEST), Palermo, Italy; ^7^Department of Biomedicine, Neuroscience and Advanced Diagnostic, Ophthalmology Section, University of Palermo, Palermo, Italy; ^8^Department of Translational Surgery and Medicine, Eye Clinic, University of Florence, Florence, Italy; ^9^Agenzia Regionale di Sanità Della Toscana, Florence, Italy; ^10^Regional Pharmacovigilance Center of Lombardy, Milan, Italy; ^11^Department of Epidemiologic Observatory, Health Department of Sicily, Palermo, Italy; ^12^Sicilian Regional Centre of Pharmacovigilance, Servizio 7-Farmaceutica, Health Department of Sicily, Palermo, Italy; ^13^Department of Health Sciences, “Magna Graecia” University of Catanzaro, Catanzaro, Italy; ^14^A.O.U. “Mater Domini”, Catanzaro, Italy

## Abstract

**Purpose:**

To describe intravitreal anti-VEGF drug and dexamethasone use in four Italian regions.

**Methods:**

Four regional claims databases were used to measure drug prevalence, compare dosing intervals to those recommended in the summary of product characteristics (SPC), and identify switchers. Bilateral treatment and diabetic macular edema (DME) coding algorithms were validated, linking claims with a sample of prospectively collected ophthalmological data.

**Results:**

Overall, 41,836 patients received ≥1 study drug in 2010–2016 (4.8 per 10,000 persons). In 2016, anti-VEGF drug use ranged from 0.8 (Basilicata) to 5.7 (Lombardy) per 10,000 persons while intravitreal dexamethasone use ranged from 0.2 (Basilicata) to 1.4 (Lombardy) per 10,000 persons. Overall, 40,815 persons were incident users of study drugs. Among incident users with ≥1 year of follow-up (*N* = 30,745), 16.0% (*N* = 4,890) had only one pharmacy claim, especially for ranibizumab (60.9%). Switching occurred in 8.0% of users with ≥2 pharmacy claims (*N* = 33,637). The algorithms had an accuracy of 83.8 (95% CI: 79.7–87.3) concerning bilateral treatment and 72.3% (95% CI: 67.5–76.8) for DME.

**Conclusion:**

Study drug use increased over time in Lombardy, Basilicata, Calabria, and Sicily, despite a large heterogeneity in prevalence of use across regions. Drug treatment appeared to be partly in line with SPC, suggesting that improvement in clinical practice may be needed to maximize drug benefits.

## 1. Introduction

The most common neovascular diseases responsible for visual impairment are age-related macular degeneration (AMD), diabetic macular edema (DME), retinal vein occlusion (RVO), and choroidal neovascularization secondary to pathological myopia (mCNV) [1, 2]. Intravitreal anti-vascular endothelial growth factor (anti-VEGF) drugs and dexamethasone have significantly improved clinical outcomes in these diseases [3]. The intravitreal anti-VEGF drugs pegaptanib, ranibizumab, and aflibercept are indicated in AMD, DME, RVO, and mCNV [4–6], while intravitreal bevacizumab is widely used off-label for AMD and DME [7]. Two glucocorticoid drugs, dexamethasone and fluocinolone acetonide intravitreal implants, are also approved for DME [8, 9].

The National Institute of Health and Clinical Excellence (NICE) guidance recommends first-line use of ranibizumab and aflibercept in AMD and DME. According to the summary of product characteristics (SPC), the induction phase for aflibercept and ranibizumab for all retinal diseases except mCNV is initially similar, with some slight differences: one monthly injection for three or five consecutive months, respectively [5, 6]. After the induction phase, the maintenance phase involves two different recommended treatment regimens: the treat-and-extend approach involves increasing or decreasing intervals between monthly injections by two weeks, while the pro re nata (PRN) approach involves one injection as needed. Pegaptanib injections should be administered once every six weeks [4]. Whatever the treatment regimen used, the interval between two consecutive injections in the same eye should be at least four weeks [5, 6]. The treatment regimen for the dexamethasone implant is simpler than that for intravitreal anti-VEGF drugs: one implant is inserted approximately every six months for the treatment of either DME or RVO [8]. Further information on all therapeutic particulars for anti-VEGF drugs and dexamethasone is available in Supplementary [Supplementary-material supplementary-material-1].

To date, it is not known how intravitreal drugs are used in Italian clinical practice. The primary objective of this study was to describe the utilization pattern of these drugs in Italy while the secondary objective was to measure the diagnostic performance of algorithms to identify bilaterally treated patients and DME in claims databases.

## 2. Methods

### 2.1. Data Source

A retrospective, drug utilization study was conducted using fully anonymized data from the claims databases of Lombardy, Sicily, Calabria, and Basilicata regions from 2010 to 2016 (Supplementary [Supplementary-material supplementary-material-1]). The national demographic data published by the Italian Institute of Statistics was used to calculate the crude yearly number of inhabitants in each region, i.e., the underlying population in the catchment areas [10].

The four databases used were claims databases linked through a unique patient identifier: a demographic registry, containing demographic patient information, hospital discharge diagnosis database, emergency department admissions database, and diagnostic and laboratory tests ordered. The Anatomical Therapeutic Chemical (ATC) classification system is used to code drug information on drugs. Diagnoses are coded using the International Classification of Diseases, 9th Revision, Clinical Modification (ICD-9 CM) [11].

To complement the claims data, clinical data was collected prospectively in three large ophthalmological centres in Sicily using a case report form (CRF). The CRF contains a range of clinical information related to the ophthalmologic visit for each patient. Information was collected at the first visit to the clinic and at each of the following visits, including check-up visits or visits for drug administration. CRF data was considered as the gold standard given that data collection was carefully curated specifically for research purposes.

### 2.2. Study Drugs

The study drugs of interest were dexamethasone intravitreal implant (ATC: S01BA01), ranibizumab (ATC: S01LA04), aflibercept (ATC: S01LA05), and pegaptanib (ATC: S01LA03). Due to the difficulty of tracing the off-label intravitreal use of bevacizumab using claims databases, this drug was not included. As fluocinolone acetonide was marketed in 2017, this could not be identified using the data available. The date of the first intravitreal drug dispensed during the study period was considered as the index date. Incident drug users were identified as patients having at least one-year intravitreal anti-VEGF or dexamethasone-free period prior to the index date.

### 2.3. Study Population

The inclusion criteria were as follows: patients with at least one year of continuous database history in the claims databases having at least one pharmacy claim for the study drugs during the observation years. Patients were followed until death, disenrollment from the local healthcare system, or end of the study, whichever occurred first.

### 2.4. Data Analysis

Descriptive statistics were reported as medians, along with interquartile range (IQR), or absolute frequency and percentages, for continuous and categorical variables, respectively. The crude yearly prevalence of use, along with 95% confidence intervals (CIs), of an intravitreal anti-VEGF drug as a class and by individual drug and dexamethasone was calculated for each region and calendar year. The yearly frequency of individual drug use over the study period was also estimated as percentage of ranibizumab, aflibercept, pegaptanib, or dexamethasone users on the total number of any intravitreal drug users.

Incident users of study drugs were described in terms of demographics and clinical characteristics such as comorbidities (identified any time prior to the index date), Charlson Comorbidity Index (CCI) [11], concomitant drug use, and number of drugs dispensed (identified within 90 days prior to the index date). For incident users having at least one year of follow-up after cohort entry, the time gap in days between two consecutive pharmacy claims was calculated and was depicted graphically by means of box plots. Since Lombardy regional claims database also contains the date of the intravitreal injection administration, recorded as a procedure code, sensitivity analyses were conducted in this catchment area to ascertain to what degree the drug dispensing date coincided with the drug administration date. Furthermore, a switching pattern was investigated among incident study drug users in the first year of treatment. Switching was defined as the first presence of a pharmacy claim for a study drug other than the index drug occurring within more than 25 days of the index date.

Patients with pharmacy claims less than 25 days apart were considered to be bilaterally treated, based on anti-VEGF SPC recommendations [5, 6]. The dexamethasone SPC does not specifically recommend a minimum period to receive implants in both eyes, apart from not recommending same-day treatment in both eyes. However, based on clinical experience, re-treatment with dexamethasone within less than 25 days is likely to indicate bilateral treatment.

Among incident users of aflibercept and ranibizumab who did not switch drugs in the first year of therapy, the number of patients who were given only 3 pharmacy claims during one year of follow-up, i.e., the minimum induction phase as recommended by the SPC, was identified. The underlying rationale is that discontinuing treatment before a subsequent treat-and-extend or PRN phase of treatment is potentially inappropriate. Pegaptanib and dexamethasone users were excluded from this analysis since the SPC does not recommend an induction phase. Finally, among incident users of any intravitreal drug with one year of follow-up, the number of patients who were given only one pharmacy claim was calculated.

Since bilateral treatment or indication of drug use was not recorded in claims data, proxy algorithms are required to identify such clinical features: claims data from Sicily were linked with prospective clinical data where the information about these clinical features was available (i.e., the gold standard). The following three algorithms were applied: (1) bilateral treatment: in line with the SPC, anti-VEGF users were considered to be bilaterally treated if the interval between two claims for anti-VEGF drugs was less than 25 days; (2) DME (narrow definition): DME was identified based on (a) a diabetes diagnosis in a hospital discharge record or (b) at least two pharmacy claims for antidiabetic drugs and a diagnosis of DME in a hospital discharge record; (3) DME (broad definition): DME was defined based on (a) a diabetes diagnosis in a hospital discharge record or (b) at least two pharmacy claims for antidiabetic drugs. The diagnostic and predictive accuracy of each claims-based algorithm was evaluated by calculating the sensitivity, specificity, overall accuracy, positive predictive value (PPV), and negative predictive value (NPV) with respective 95% CIs [12].

All statistical analyses were performed using SAS Release 9.4 (SAS Institute, Cary, NC, USA) and SPSS/PC, Version 15 (SPSS Inc., Chicago, Illinois, USA). Plots were produced using SAS and Cytoscape software (http://www.cytoscape.org/).

## 3. Results

### 3.1. Prevalence, Incidence of Use, and Characterization of Incident Users of Study Drugs

**Figure 1:**
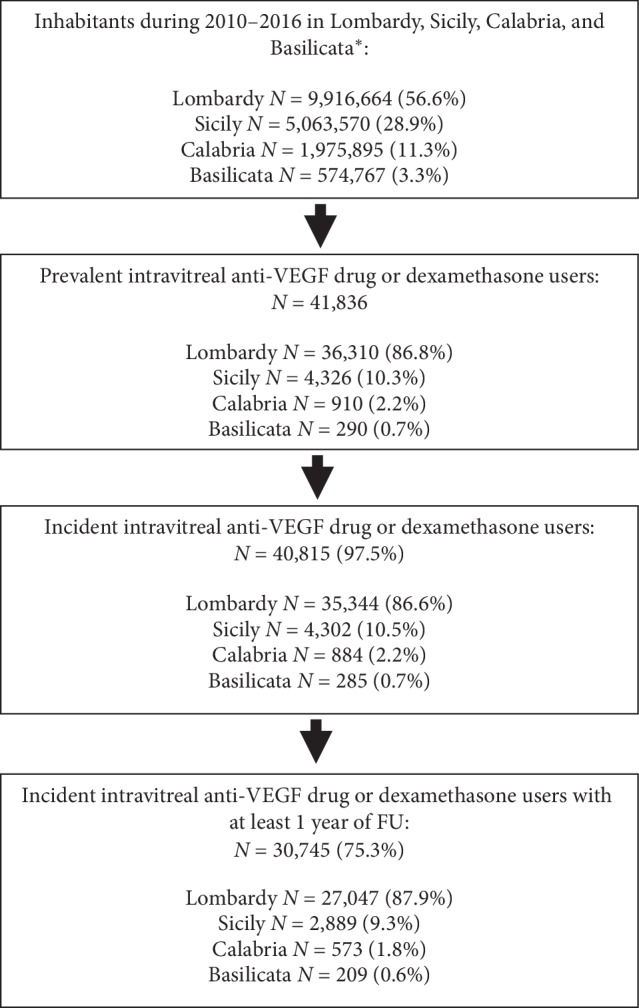
Identification of incident users of intravitreal anti-VEGF drugs or dexamethasone. Footnote: anti-VEGF: vascular endothelial growth factor; ID: Index Date (i.e., date of the first anti-VEGF or intravitreal dexamethasone dispensing during the study years). ^*∗*^Data availability ranged from 2010 to 2016 in Lombardy, from 2012 to 2015 in Calabria, and from 2012 to 2016 in Sicily and Basilicata. The crude numbers of inhabitants of each region used to calculate the mean yearly populations were based on official demographic data published by the Italian National Institute of Statistics

The catchment areas covered approximately 18 million persons, i.e., almost a third of the Italian population. From 2010 to 2016, at least one study drug was dispensed to 4.8 per 10,000 persons (Figure 1).

**Figure 2:**
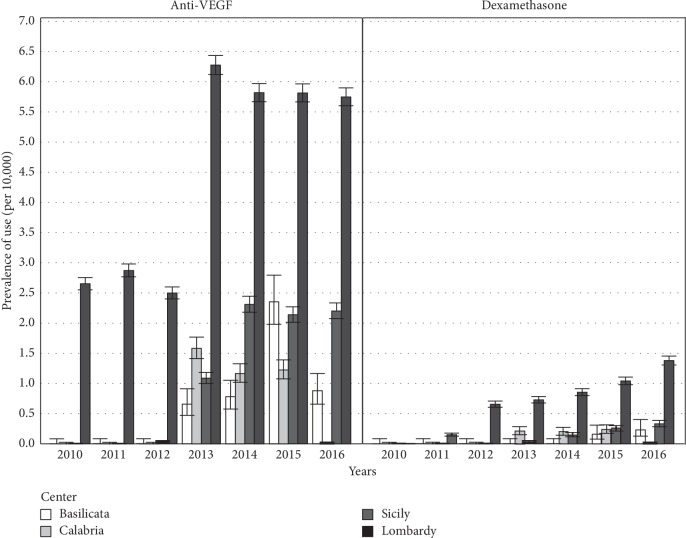
Yearly prevalence of use per 10,000 persons of intravitreal anti-VEGF or dexamethasone in the general population, grouped by region and calendar years (2010–2016). Error bars represent the Wilson score 95% confidence interval (with continuity correction) around prevalence estimates.

Stratifying by region, the prevalence of anti-VEGF drug users in the general population was much higher in Lombardy than other regions, ranging from 2.65 (95% CI: 2.55–2.75) anti-VEGF users per 10,000 persons in 2010 to 5.74 (95% CI: 5.60–5.89) anti-VEGF users per 10,000 persons in 2016. In the other regions, the prevalence was lower, particularly in Basilicata (Figure 2).

Between 2012 and 2013, the anti-VEGF drug use more than doubled in Lombardy and Sicily, the only two regions where data for these years was available (0.02 vs. 1.08 anti-VEGF users per 10,000 persons in Sicily and 2.49 vs. 6.27 anti-VEGF users per 10,000 persons in Lombardy). The yearly prevalence of dexamethasone use showed an increasing trend which was most clearly seen in Lombardy, starting at 0.15 (95% CI: 0.12–0.17) dexamethasone users per 10,000 persons to 1.37 (95% CI: 1.30–1.45) per 10,000 persons in 2016.

Among the prevalent users of any intravitreal drug, the yearly distribution of drug use changed over time: ranibizumab gradually decreased from 90% in 2010 to 53% in 2016, while aflibercept use increased from approximately 7% in 2014 to 30% in 2016. Pegaptanib gradually fell into disuse. Dexamethasone use increased from 10% in 2013 to 18% in 2016 (Supplementary [Supplementary-material supplementary-material-1]).

Among the users of anti-VEGF drugs, 40,815 (97.5% of patients with at least one pharmacy claim for a study drug) were incident users. The most frequently dispensed drug at cohort entry was ranibizumab (*N* = 30,298; 74.0% of new intravitreal drug users) while the least common was pegaptanib (*N* = 525; 1.28% of new intravitreal drug users) (Table 1).

**Figure 3:**
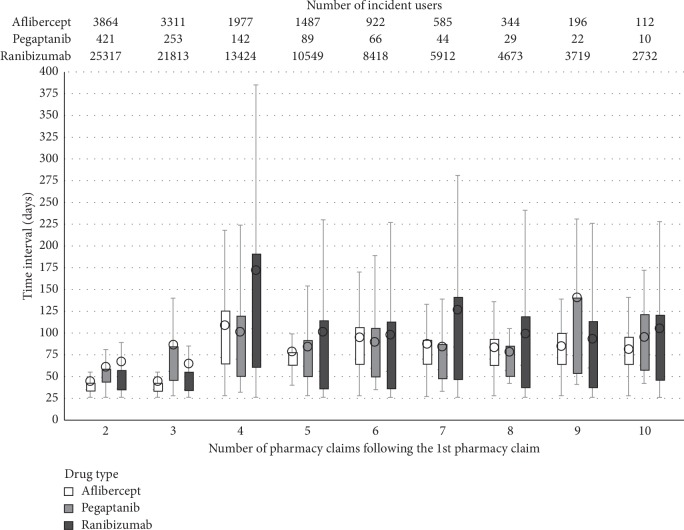
Distribution of the number of days between intravitreal anti-VEGF pharmacy claims among incident users, by drug dispensed at cohort entry. The solid line inside each box represents the median value whereas each circled dot represents the mean value.

Overall, 53.5% (*N* = 25,317) of the incident ranibizumab users and 82.4% (*N* = 3,864) of the incident aflibercept users with at least 2 pharmacy claims had a median treatment gap of 1 month (IQR: 34–56), in line with the SPC. This distribution remained similar among ranibizumab and aflibercept users between their second and third doses of anti-VEGF (Figure 3).

The IQR of these initial treatment gaps was relatively narrow, suggesting low variability. From the fourth pharmacy claim onwards, the median treatment gap was much higher, with a markedly wider IQR (ranibizumab: 105 (IQR: 60–190) days, aflibercept: 72 (IQR: 63–125) days). The median and IQR values of the treatment gap remained constant from the fifth pharmacy claim onward. The sensitivity analysis using the drug administration date in place of drug dispensing date in the Lombardy claims database (Supplementary [Supplementary-material supplementary-material-1]) confirmed the main results (Supplementary Materials [Supplementary-material supplementary-material-1] and [Supplementary-material supplementary-material-1]).

**Figure 4:**
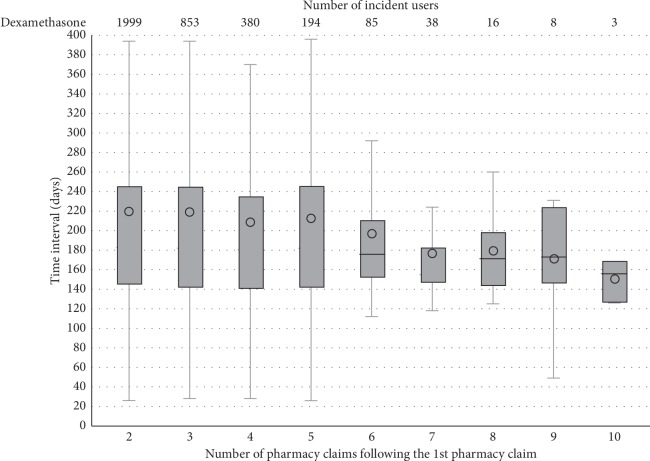
Distribution of the number of days between intravitreal dexamethasone pharmacy claims among incident users. The solid line inside each box represents the median value whereas each circled dot represents the mean value.

Dexamethasone users were found to generally have a pharmacy claim every 180 days (Figure 4).

The median time between the first and the second dexamethasone pharmacy claim was 183 days (IQR: 145–245 days); this IQR suggests that some users are retreated just before or little later (up to approximately 40 days earlier or 60 days later) than expected based on SPC. The same median treatment gap and interquartile range were observed until the fifth pharmacy claim.

**Figure 5:**
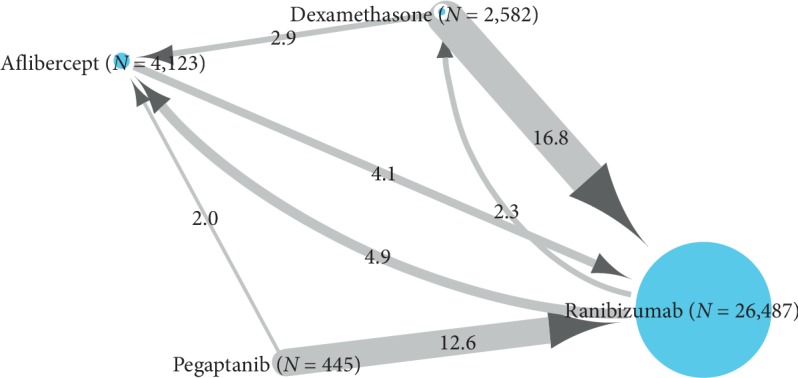
Switching pattern among incident users with at least two pharmacy claims within the first year of treatment after cohort entry. Percentages are shown on the arrows.

Therapeutic switching occurred in 8.0% (*N* = 2,701) of incident users with at least two pharmacy claims during the first year of treatment (*N* = 33,637) (Figure 5). Switching was most frequently observed from dexamethasone to ranibizumab (16.8%).

In the first year of treatment, *N* = 6,601 (16.0% of all incident users) were likely treated in both eyes, since they had consecutive pharmacy claims within 25 days of each other. Users of dexamethasone were most commonly bilaterally treated based on our algorithm (33.3% of all incident dexamethasone users), followed by ranibizumab users (14.6% of all incident ranibizumab users).

Among incident users of intravitreal anti-VEGF drugs or dexamethasone with ≥1 year of follow-up (*N* = 30,745), 16.0% (*N* = 4,890) had only one pharmacy claim. Stratifying by a single drug, ranibizumab users were most commonly dispensed one drug only (*N* = 2,970; 60.9% of patients with one pharmacy claim). Among the incident users of ranibizumab who did not switch in the first year of treatment (*N* = 23,037), 7,896 (34.2%) were given only the minimum loading dose of 3 injections. Among 1,978 incident users of aflibercept who did not switch in the first year of treatment, 550 (27.8%) received the minimum loading dose of 3 injections.

The identification of bilaterally treated patients or those affected with DME could be carried out in 380 Sicilian patients identified in the claims data for whom prospectively collected clinical data was available and could be linked to the claims data. For the detection of bilateral treatment, the algorithm achieved an overall accuracy of 83.8% (95% CI: 79.7–87.3). Concerning the detection of DME, the algorithm having a narrow definition achieved a high overall accuracy of 72.3% (95% CI: 67.5–76.8) (Supplementary [Supplementary-material supplementary-material-1]). Both these results suggest a good overall identification of patients.

## 4. Discussion

To date, this is the only drug utilization study exploring the real-world pattern of intravitreal anti-VEGF drug and dexamethasone use in Italy, using data from four large Italian regions covering approximately 30% of the Italian population. The main finding from this study was the increasing use of intravitreal drugs in Italy. The prevalence of anti-VEGF drug use was generally higher than the use of dexamethasone, with this being most evident in Lombardy, where after doubling abruptly in 2013, it remained constant until 2016. A similar increase in intravitreal anti-VEGF drug use was seen at the national level in the Italian National Report on Medicines Use (OsMed), where the transition between 2012 and 2013 was marked by an abrupt and large increase in anti-VEGF use of over 116% [13, 14]. We hypothesize that this sudden increase is related to a regulatory event in 2012 when the Italian Drug Agency removed the anti-VEGF drug bevacizumab from the list of nationally reimbursed drugs used in off-label ways for ophthalmological indications after the European Medicines Agency expressed concerns about its safety [15]. Indeed, over the study period, we were able to quantify the use of exclusively off-label bevacizumab users ranging from only 1% in Sicily to 7% in Lombardy.

The high prevalence of anti-VEGF use in Lombardy is in line with the 2017 regional Italian National Report on Medicines Use, where intravitreal anti-VEGF drug use was 26% higher than the national average, while, in Sicily and Calabria, anti-VEGF drug use was 11% and 29% lower than the national average, respectively [16–18]. A possible explanation for the higher use of intravitreal drugs in Lombardy concerns the extent of claims data captured, which is greater in Lombardy. The higher number of regional claims captured in Lombardy may be due to the slightly different, simpler reimbursement processes, as a result of which, more claims are captured. In addition, it is possible that the higher prevalence of study drugs in Lombardy may also be due to a greater number of specialized ophthalmology centres compared to other regions. To quantify the potential extent of the underestimation of pharmacy claims, we compared the number of Sicilian pharmacy claims in 2016 for ranibizumab and aflibercept to prescriptions recorded in two Sicilian drug monitoring registers for these drugs, where clinicians are obliged by law to record all such prescriptions. This comparison was only made in Sicily as data from these monitoring registries was only available in this region. Pharmacy claims for ranibizumab and aflibercept were found to account only for 58% of the pharmacy claims recorded in the Sicilian registries. This underreporting in claims may occur because it is not mandatory to file a drug claim in order for drugs to be reimbursed.

The main finding regarding the intravitreal drug use patterns was the varied treatment regimen in terms of the interval between one pharmacy claim and another. The wide IQRs suggest heterogeneity in the timing of drug administration. For example, ranibizumab injections commonly occurred approximately monthly up to the third pharmacy claim, potentially indicating loading doses. From the fourth pharmacy claim onward, ranibizumab was likely used in a PRN or treat-and-extend approach. This would be largely in line with the SPC. The wide treatment intervals may reflect personalized treatment, incorrect use, or delays unrelated to clinical treatment, such as becoming bedridden or otherwise very disabled, as well as logistical difficulty in going to the treatment centre [19].

Therapeutic switching occurred in a relatively low proportion of incident users. The highest proportion of switching was observed from dexamethasone to ranibizumab (17%). The claims do not provide detail on the reasons for switching, but in clinical practice, this generally occurs due to therapeutic inefficacy.

A relatively low proportion of incident intravitreal drug users was found to be bilaterally treated, at 16.0% (*N* = 6,601). At the moment of writing, there are no similar studies to which the present study can be compared. Both ranibizumab and dexamethasone were the most common drugs utilized in patients whose drug dispensing patterns suggested bilateral treatment. This is supported by the high prevalence of DME among neovascular diseases, where bilateral use of anti-VEGF and/or dexamethasone injections is common [20].

Among incident users with ≥1 year of observation time, 16.0% of users received only 1 pharmacy claim. This was seen particularly for ranibizumab and dexamethasone users. The reasons may be similar to those for excessively short or long treatment intervals: adverse drug reactions, nonresponse (e.g., in advanced state of disease), or other situations related to older age (e.g., death or problems related to the poor or difficult mobility of these subjects) which could prompt suspension of treatment or a diagnosis which requires nonpharmacologic treatment.

The present study must be interpreted in light of its limitations: results cannot be generalized to the whole Italian population due to large regional differences [21]. In addition, drug utilization may be underreported in some regions, although broadly in line with the National Report on Medication Use. Furthermore, we were not able to evaluate the economic impact of the use of these drugs as we did not have this information. Finally, while we were able to validate two important aspects of ophthalmological treatment, i.e., bilateral treatment and DME diagnosis, claims data generally lack clinical detail, which, as a result, could not be reported.

## 5. Conclusions

Intravitreal drug use increased over time in four large Italian regions with a notable heterogeneity across regions. Treatment regimens are not always in line with the respective SPCs, particularly concerning dexamethasone. The use of algorithms for the identification of DME and bilateral patients shows that claims databases are a valuable source of real-world data for postmarketing assessment of anti-VEGF drugs.

## Figures and Tables

**Table 1:** Characteristics of incident anti-VEGF/dexamethasone users, stratified by drug dispensed at the index date.

	Ranibizumab *N* = 30,298 (%)	Aflibercept *N* = 4,689 (%)	Pegaptanib *N* = 525 (%)	Dexamethasone *N* = 5,303 (%)	Overall *N* = 40,815 (%)
Sex					
Female	16,735 (55.2)	2,594 (55.3)	272 (51.8)	2,442 (46.0)	22,043 (54.0)
Male	13,563 (44.8)	2,095 (44.7)	253 (48.2)	2,861 (54.0)	18,772 (46.0)

Age (years), median (IQR)	73.7 (67.0–82.0)	76.1 (71.0–83.0)	78.0 (73.0–83.0)	70.0 (63.0–78.0)	73.5 (67.0–82.0)

Age classes					
<65	5,434 (17.9)	518 (11.0)	30 (5.7)	1,462 (27.6)	7,444 (18.2)
65–74	8,510 (28.1)	1,201 (25.6)	122 (23.2)	1,745 (32.9)	11,578 (28.4)
75–84	11,842 (39.1)	2,116 (45.1)	267 (50.9)	1,699 (32.0)	15,924 (39.0)
≥85	4,512 (14.9)	854 (18.2)	106 (20.2)	397 (7.5)	5,869 (14.4)

Centre					
Lombardy	26,270 (86.7)	3,790 (80.8)	508 (96.8)	4,776 (90.1)	35,344 (86.6)
Sicily	3,100 (10.2)	811 (17.3)	10 (1.9)	381 (7.2)	4,302 (10.5)
Calabria	673 (2.2)	80 (1.7)	7 (1.3)	124 (2.3)	884 (2.2)
Basilicata	255 (0.8)	8 (0.2)	—	22 (0.4)	285 (0.7)

FU (years), median (Q1-Q3)	2.6 (1.0–4.0)	0.9 (0–2.0)	4.3 (3.0–6.0)	1.9 (1.0–3.0)	2.3 (1.0–3.0)

Comorbidities					
Cardio- and cerebrovascular disease					
Hypertension	23,041 (76.0)	3,717 (79.2)	438 (83.4)	4,046 (76.3)	31,242 (76.5)
Heart failure	1,678 (5.5)	299 (6.4)	33 (6.3)	344 (6.5)	2,354 (5.8)
Arrhythmias	3,268 (10.8)	619 (13.2)	83 (15.8)	545 (10.3)	4,515 (11.1)
Venous or arterial TE	292 (1.0)	44 (0.9)	4 (0.8)	51 (1.0)	391 (1.0)
Ischemic heart disease	2,627 (8.7)	439 (9.4)	66 (12.6)	535 (10.1)	3,667 (9.0)
Stroke and TIA	4,208 (13.8)	744 (15.8)	124 (23.6)	873 (16.4)	6,029 (14.7)

Metabolic disease					
Dyslipidemia	13,410 (44.3)	2,133 (45.5)	236 (45.0)	2,352 (44.4)	18,131 (44.4)
Diabetes mellitus	9,918 (32.7)	1,160 (24.7)	101 (19.2)	1,688 (31.8)	12,867 (31.5)

Neurodegenerative disorders					
Parkinson's disease	686 (2.3)	148 (3.2)	15 (2.9)	122 (2.3)	971 (2.4)
Dementia	207 (0.7)	35 (0.7)	4 (0.8)	24 (0.5)	270 (0.7)

Other diseases					
CKD	768 (2.5)	133 (2.8)	16 (3.0)	207 (3.9)	1124 (2.8)
COPD	576 (1.9)	194 (4.1)	5 (1.0)	102 (1.9)	877 (2.1)
Glaucoma	1,767 (5.8)	205 (4.4)	30 (5.7)	283 (5.3)	2,285 (5.6)
Cancer	5,581 (18.4)	1,038 (22.1)	86 (16.4)	900 (17)	7,605 (18.6)

Comorbidities Charlson's index					
0	16,271 (53.7)	2,628 (56.0)	304 (57.9)	2,894 (54.6)	22,097 (54.1)
1	1,739 (5.7)	370 (7.9)	67 (12.8)	269 (5.1)	2,445 (6.0)
2	1,823 (6.0)	397 (8.5)	37 (7.0)	366 (6.9)	2,623 (6.4)
3	6,027 (19.9)	767 (16.4)	71 (13.5)	950 (17.9)	7,815 (19.1)
≥4	4,438 (14.6)	527 (11.2)	46 (8.8)	824 (15.5)	5,835 (14.3)

Number of drugs^*∗*^					
0	3,401 (11.2)	559 (11.9)	31 (5.9)	561 (10.6)	4,552 (11.2)
1–3	10,660 (35.2)	1,609 (34.3)	140 (26.7)	1,932 (36.4)	14,341 (35.1)
4–6	9,085 (30.0)	1,435 (30.6)	195 (37.1)	1,555 (29.3)	12,270 (30.1)
>6	7,152 (23.6)	1,086 (23.2)	159 (30.3)	1,255 (23.7)	9,652 (23.6)

CDK: chronic kidney disease; COPD: Chronic obstructive pulmonary disease; FU: follow-up; IQR: interquartile range (i.e., 1st - 3rd quartiles); TE: thromboembolism; TIA: transient ischemic attack; VEGF: vascular endothelial growth factor. ^*∗*^Within 90 days prior to the index date.

## Data Availability

The data used to support the findings of this study are not available from the corresponding author upon request because of data use agreement restrictions made with the data providers.

## Supplementary Materials

Supplementary Materials Supplementary Material 1: anti-VEGF drugs and dexamethasone available on the market in Italy for the treatment of neovascular retinal diseases during the study period. Supplementary Material 2: chart of Italy. Supplementary Material 3: yearly distribution of an intravitreal anti-VEGF drug or dexamethasone users among users of any intravitreal drug from 2010 to 2016, stratified by every single drug. Error bars represent the Wilson score 95% confidence interval (with continuity correction) around prevalence estimate. Supplementary Material 4: identification of anti-VEGF drug and dexamethasone users in Lombardy for whom the procedure dates of intravitreal injections were available. Supplementary Material 5: distribution of the number of days among incident users receiving consecutive intravitreal injections of anti-VEGF drugs using procedure dates, stratified by study drug dispensed at cohort entry. The solid line inside each box represents the median value whereas each circle dot represents the mean value. Supplementary Material 6: distribution of the number of days among incident users receiving consecutive intravitreal implants of dexamethasone using procedure dates, stratified by study drug dispensed at cohort entry. The solid line inside each box represents the median value whereas each circle dot represents the mean value. Supplementary Material 7: validation measures to ascertain the correct identification of bilateral treatment and diabetic macular edema.
