# Supplementary material for: How Have Intravitreal Anti-VEGF and Dexamethasone Implant Been Used in Italy? A Multiregional, Population-Based Study in the Years 2010–2016
Source: Biomed Res Int. 2020 Jan 9;2020:7582763. doi: 10.1155/2020/7582763 (PMC6977336; doi:10.1155/2020/7582763)
Supplement: Supplementary Materials — Supplementary Material 1: anti-VEGF drugs and dexamethasone available on the market in Italy for the treatment of neovascular retinal diseases during the study period. Supplementary Material 2: chart of Italy. Supplementary Material 3: yearly distribution of an intravitreal anti-VEGF drug or dexamethasone users among users of any intravitreal drug from 2010 to 2016, stratified by every single drug. Error bars represent the Wilson score 95% confidence interval (with continuity correction) around prevalence estimate. Supplementary Material 4: identification of anti-VEGF drug and dexamethasone users in Lombardy for whom the procedure dates of intravitreal injections were available. Supplementary Material 5: distribution of the number of days among incident users receiving consecutive intravitreal injections of anti-VEGF drugs using procedure dates, stratified by study drug dispensed at cohort entry. The solid line inside each box represents the median value whereas each circle dot represents the mean value. Supplementary Material 6: distribution of the number of days among incident users receiving consecutive intravitreal implants of dexamethasone using procedure dates, stratified by study drug dispensed at cohort entry. The solid line inside each box represents the median value whereas each circle dot represents the mean value. Supplementary Material 7: validation measures to ascertain the correct identification of bilateral treatment and diabetic macular edema. [file 7582763.f1.pdf]

Supplemental Material 1.

Active substance	Brand name	MA date	Indication	Summary of product characteristics recommendations
**Aflibercept**	Eylea®	2013	nAMD	Three monthly injections, followed by 1 injection every 2 months. After the first 12 months of treatment, the treat-and-extend approach may be adopted.
			Branch or central RVO	Monthly injections. After the first 3 injections, the treat-and-extend approach may be adopted.
			DME	Five monthly injections, followed by 1 injection every 2 months. After the first 12 months of treatment, the treat-and-extend approach may be adopted.
			CNV	Injections as needed (1 injection may be sufficient)
**Ranibizumab**	Lucentis®	2007	nAMD<br>Branch or central RVO<br>DME	Three or more monthly injections, followed by the treat-and-extend approach.
			CNV	Injections as needed (1 injection may be sufficient).
**Pegaptanib**	Macugen®	2007	nAMD	One injection every 6 weeks.
**Dexamethasone**	Ozurdex®	2011	Branch or central RVO<br>DME<br>uveitis	One injection every 6 months.<br>Administration to both eyes concurrently is not recommended.
**Bevacizumab**	Avastin®	2005	nAMD	Off-label use.

Supplementary Material 2.

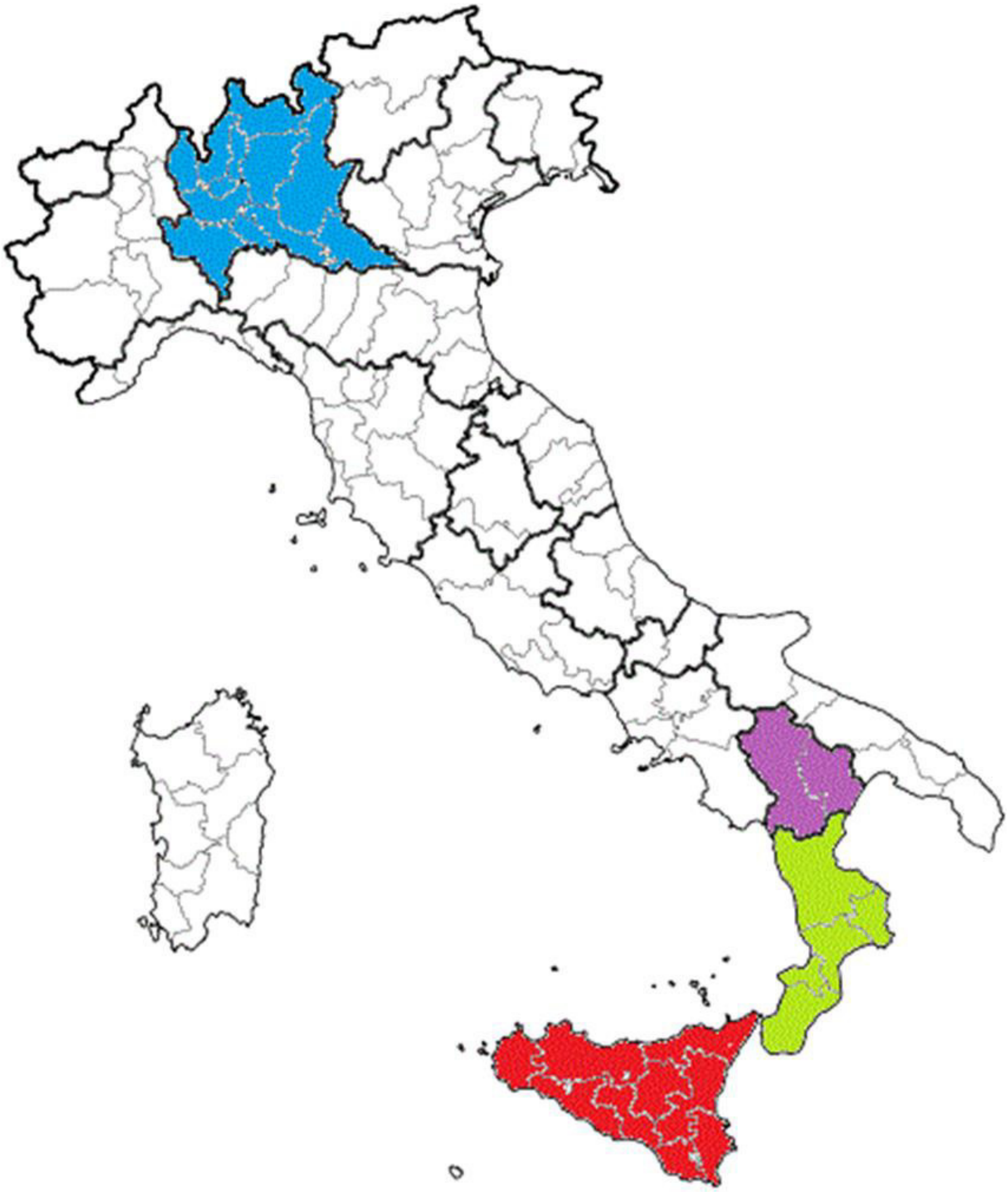

Supplemental Material 3.

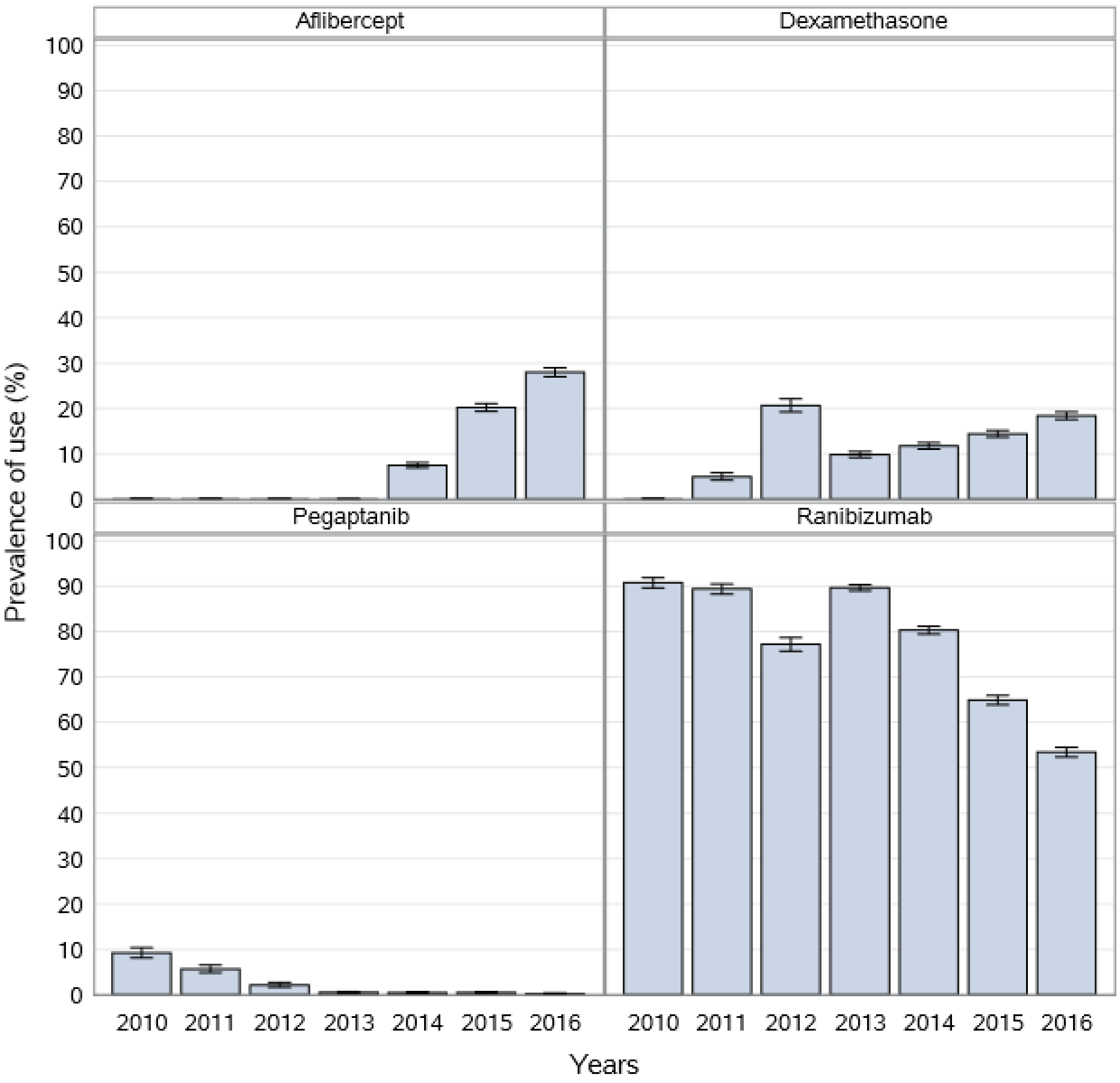

Supplemental Material 4.

Persons with at least one year of database history in the study period (2010-2016) in Lombardy Region and at least one anti-VEGF drug or dexamethasone dispensing:
N= 36,315

↓

Persons with at least one procedure of intravitreal injection of the study drugs in Lombardy Region in the study period:
N= 34,736 (95.7%)

↓

Incident anti-VEGF or dexamethasone users with at least one procedure of intravitreal injection:
N= 33,875 (97.5%)

↓

Incident anti-VEGF or dexamethasone users with at least one year of follow-up after ID, not potentially bilateral treated and with no switch within the first year of treatment:
N= 23,501 (69.4%)

Supplemental Material 5.

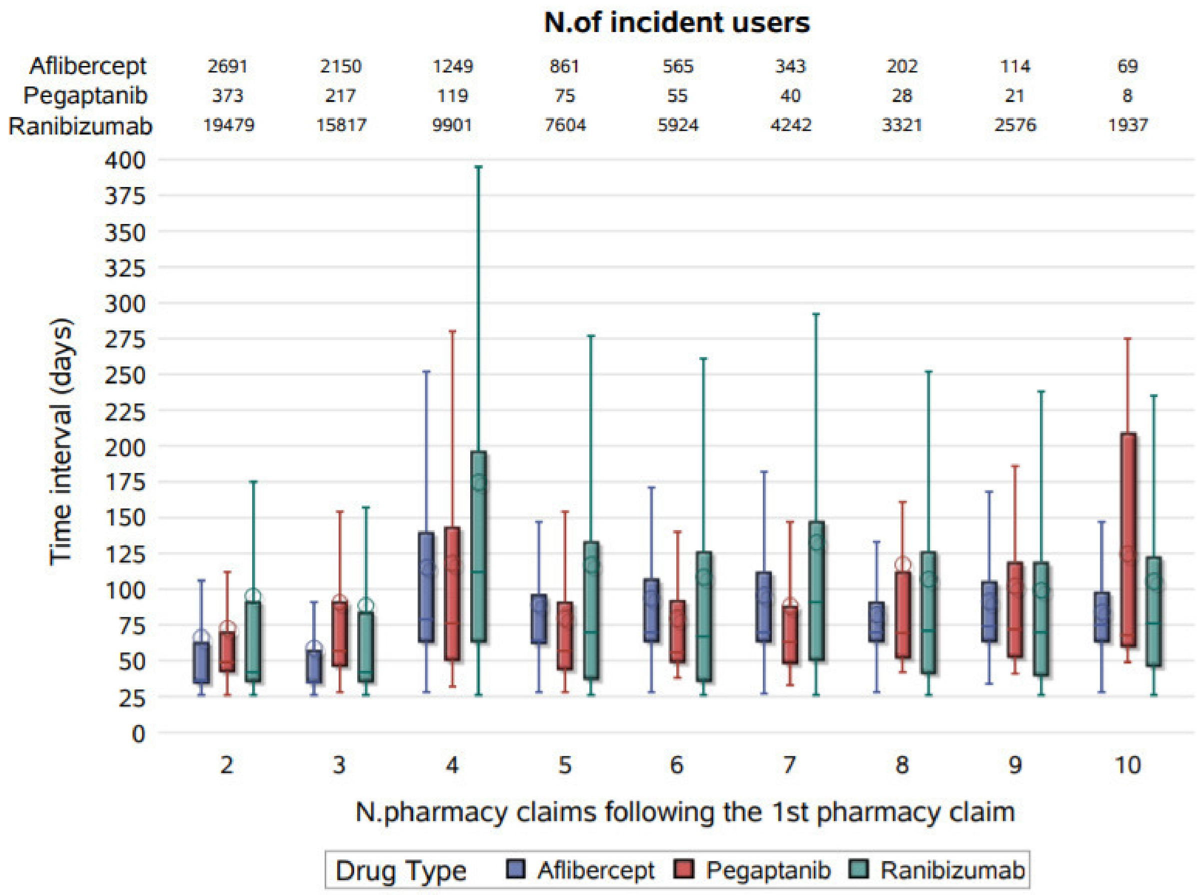

Supplemental Material 6.

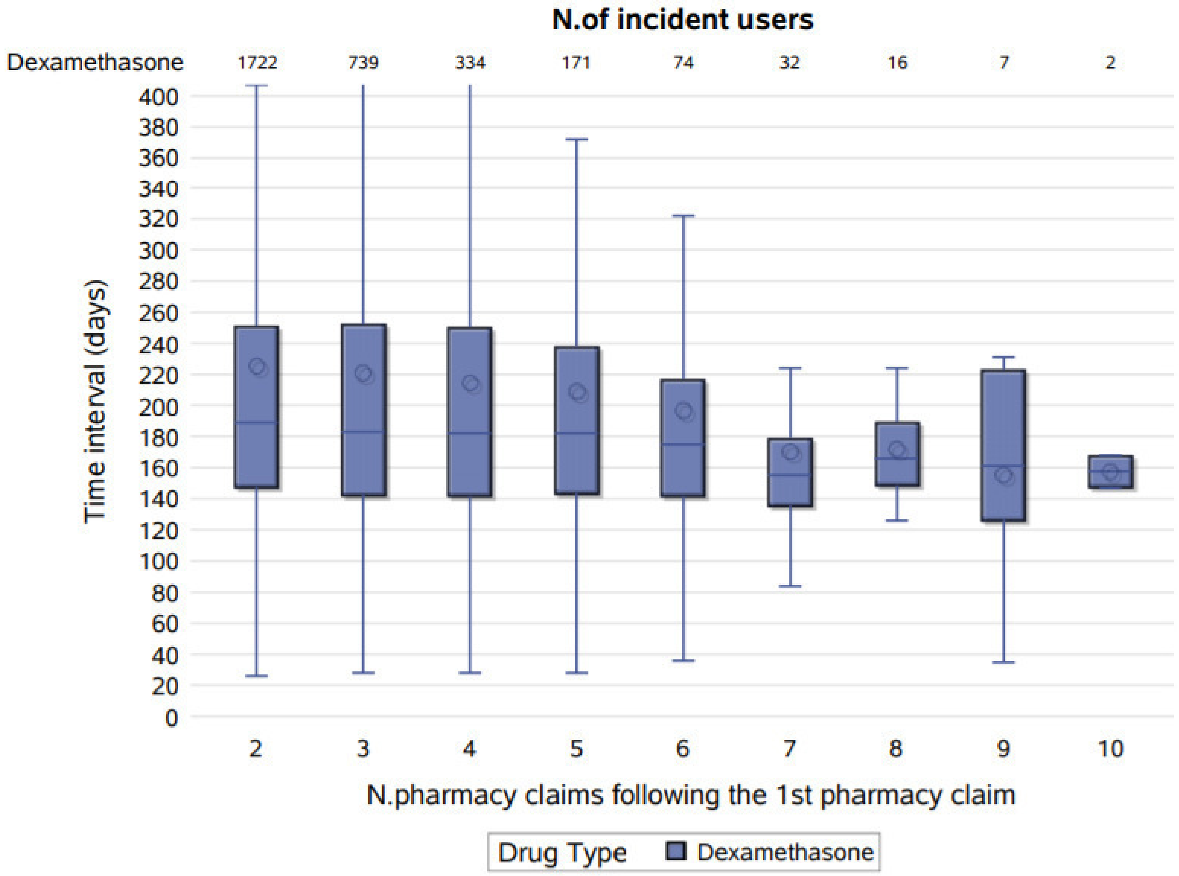

Supplemental Material 7.

		Sensitivity<br>% (95% CI)	Specificity<br>% (95% CI)	Overall accuracy<br>% (95% CI)	PPV<br>% (95% CI)	NPV<br>% (95% CI)
**Bilateral treatment**		25.3 (15.5 - 37.5)	96.0 (93.2 - 97.8)	83.8 (79.7 - 87.3)	56.6 (40.0 - 71.9)	86.0 (84.2 - 87.7)
**DME**	**Broad definition**	35.6 (30.1 - 41.5)	19.8 (12.2 - 29.5)	31.8 (27.2 - 36.8)	58.5 (54.0 - 62.9)	8.8 (6.0 - 12.9)
	**Narrow definition**	17.7(12.5 - 25.6)	98.8 (96.6 - 99.8)	72.3 (67.6 - 76.8)	88.0 (69.1 - 96.0)	71.2 (69.5 - 72.9)
